# *Oligochaeta ramosa* (Roxb.) Extract Regulates Lipid Metabolism and Exerts Hepatoprotective Effects in Cadmium-Induced Hepatic Injury in Rats

**DOI:** 10.1155/2022/2756769

**Published:** 2022-11-01

**Authors:** Saira Aslam, Fiaz-ud-Din Ahmad, Waseem Hassan, Manal Ali Buabeid, Imran Mukhtar, Muhammad Ihtisham Umar, Nihal Abdallah Ibrahim

**Affiliations:** ^1^Department of Pharmacology, Faculty of Pharmacy, The Islamia University of Bahawalpur, Bahawalpur, Pakistan; ^2^Department of Pharmacy, COMSATS University Islamabad, Lahore Campus, Lahore 54000, Pakistan; ^3^Department of Pharmacy, Fatima College of Health Sciences, Abu Dhabi, UAE; ^4^Department of Clinical Sciences, College of Pharmacy and Health Sciences, Ajman University, Ajman, UAE

## Abstract

Environmental pollutants present a potential source of toxicity when exposed to humans. The study was aimed at investigating the potential of *Oligochaeta ramosa* (Roxb.) as a hepatoprotective agent in cadmium-induced hepatotoxicity causing lipid profile disturbance. The aqueous methanolic (30 : 70 *v/v*) extract of *O. ramosa* Roxb. (AME.Or) was subjected to preliminary phytochemical analysis, whereas the antioxidant activity of its constituents was investigated by 2,2-diphenyl-1-picrylhydrazyl (DPPH) assay. The hepatoprotective and antihyperlipidemic effects of AME.Or was investigated by dividing animals into five groups (A–E). Animals were either treated with normal saline or CdCl_2_ (6.5 mg/kg, intraperitoneally) followed by treatment with silymarin (100 mg/kg), or AME.Or (200 mg/kg) and AME.Or (400 mg/kg) for consecutive three weeks. Blood samples were collected, and the serum lipid profile was assessed on the 11^th^ and 21^st^ day of treatment. Histopathological analysis was performed after euthanization. *In vitro* analysis of AME.Or revealed 64% inhibition as free radicals scavenging potential during DPPH, total phenolic content (TPC) (79.92 mgGAE/g), and total flavonoids content (TFC) (38.75 mgRE/g). The group intoxicated with CdCl_2_ showed significantly high (*p* ≤ 0.05) levels of the liver function indicators and lipid profile than in the control group. The higher dose of AME.Or (400 mg/kg) significantly decreased the aspartate aminotransferase (AST), alanine transferase (ALT), alkaline phosphatase (ALP), total bilirubin (*p* ≤ 0.001), decreased total cholesterol and triglycerides (*p* ≤ 0.01) while significantly increased high density lipoprotein (HDL; *p* ≤ 0.01) as compared to the intoxicated group. The histopathological analysis of the liver revealed signs of necrosis in the intoxicated group, while AME.Or treated groups showed marked improvement. The findings accentuate the therapeutic importance of *O. ramosa* (Roxb.) as a hepatoprotective remedy.

## 1. Introduction

*Oligochaeta ramosa* Roxb. is a straggling herb that belongs to the family Asteraceae [1]. The herb is found in the southwestern areas of Pakistan [2]. The flowering and fruiting time of *O. ramosa* is from January to March. Its leaves are obovate and its heads are ovoid with a pale-purple color. The achenes are angled and grooved with a narrow, dull-brow base [1]. The phytochemical investigations have confirmed the presence of sesquiterpenes, flavonoid (jaceosidine, apigenin, chrysoeriol, 5,7,4′-trihydroxy-3,8-dimethoxylflavone), long-chain esters, cycloartane-triterpenoids, lactone (cynaropicrin, amberen, ramosine), glycoside (Kaempferol 5-*O*-*β-D*-*gluco-pyranoside*), alkaloids, carbohydrates, tannins, saponins, anthraquinones, and steroids in *O. ramosa* Roxb. extracts [3–6].

The folkloric uses include antiemetic, antimicrobial, astringent, purgative, resolvent, antidote, laxative, and antipyretic properties [7, 8]. In trivial medicines, the herb has been used for the treatment of skin irritation, cough, external swelling, wounds, and hepatic ailments [9]. Fresh juice of *O. ramosa* Roxb. is used with *Piper nigrum* for blood purification [10]. Moreover, its aerial parts have been used for the treatment of cough, fever, and diarrhea as a bactericidal against *Helicobacter pylori* [11].

The route of cadmium (Cd) exposure plays a key role in determining the extent of cadmium absorption. Most of the Cd is exposed to the body *via* air, skin, and intestine. Factors including age, diet, lifestyle, and gender also play an important role [12]. Besides, intestinal absorption of Cd is affected by iron, vitamin D, and high dietary fiber (Flanagan et al., 1978. Around 5–9% of the total digested Cd is absorbed from the intestine where the iron deficiency causes an increase in absorption of Cd by promoting the production of divalent metal ion transport protein in a concentration dependant manner [12–15]. The skin absorbs 0.5-0.6% of the Cd in its aqueous solution. Low-cost jewelry might be a source of Cd that is absorbed through the skin [16].

Cd exposure is proven, both experimentally and epidemiologically, to cause hepatic toxicity and lipid imbalance by affecting biological processes *via* membrane damage, genetic alterations, enzyme inhibition, and electron transport disruption [17]. Cd poisoning occurs as a consequence of industrialized use as well as the presence of the element in inorganic fertilizers. Cd contamination is a major international issue since the metal is known to permeate the food chain and can be bio-accumulated, putting human lives at risk [18].

Since the liver is the key regulator of Cd metabolism and accumulation/deposition, hepatic tissues are more vulnerable to Cd-induced necrosis. It accumulates predominantly in soft tissues (particularly the liver and kidneys), due to the long biological half-life (about 20–30 years in humans) and limited rate of elimination from the body and excessive generation of ROS are the possible mechanisms of Cd-toxicity [19].

Furthermore, oxidative stress, alterations in gene expression and suppression of damaged DNA repair, interference with apoptosis and autophagy, and interaction with bio elements all are mechanisms of Cd toxicity. In hepatocytes, Cd causes oxidative stress, which leads to a variety of pathologies and consequences. Cd toxicity is linked to free radicals-induced lipid peroxidation, which causes a substantial rise in thiobarbituric acid reactive substances (TBARS) in the liver [20].

Moreover, Cd induces disruption in lipid metabolism thus the concentrations of phospholipids (PL), free fatty acids (FFA), total cholesterol (TCh), high, intermediate, low, and very-low-density lipoprotein cholesterol, and triglycerides (TG) [21].

Limited data is available on the nutritional and compositional status and bioactive potential of *O. ramosa* Roxb. However, the plant is not much known for its biological role concerning its hepatotonic properties. So, this research aimed to investigate the hepatoprotective potential of the aqueous methanolic extract of *O. ramosa* Roxb. in Wistar albino rats intoxicated with CdCl_2_.

## 2. Materials and Methods

### 2.1. Chemicals and Equipment

2,2-diphenyl-1-picrylhydrazyl (DPPH) reagent, gallic acid, rutin and silychristin (silymarin II), and methanol were purchased from Sigma Aldrich (Germany). The rotary evaporator was purchased from Buchi Laboratory Instruments (Switzerland). A hot air oven was acquired from Biobase (China).

### 2.2. Collection of Plant Material

The dried whole plant of *O. ramosa* Roxb. was procured from a local market in Bahawalpur, Pakistan. The plant was authenticated by The Department of Botany, Islamia University Bahawalpur (IUB), and a specimen was submitted to the herbarium (voucher numbers OR-WP-06-21-185) for future reference.

### 2.3. Preparation of Aqueous Methanolic Extract of *O. ramosa* (Roxb.) AME.Or

The dried whole plant of *O. ramosa* (Roxb.) was ground to a coarse powder with the commercial grinder and soaked in an aqueous methanolic (30 : 70 v/v) solution. The soaked material was filtered firstly with muslin cloth and then with Watmann's filter paper to get filtrate. This procedure was repeated thrice. Then the whole filtrate was subjected to rotary evaporation at low temperature (30–45°C) under reduced pressure (40–45Psi). A lesser viscous paste of extract was obtained from rotary evaporation, further drying was carried out in the hot air oven. A thick viscous paste of Aqueous methanolic extract of *O. ramose* (AME.Or) was obtained. At last, it was weighed for the purpose to calculate the percentage yield and stored in an air-tight container with proper labeling at −20°C.

### 2.4. *In Vitro* Phytochemical Screening

*In vitro* screening of AME.Or was carried out to confirm the presence of various secondary metabolites. Different para tests were performed to detect the presence of alkaloids (Dragendroff's test, Mayer's test, Hager's test, Wagner's test), carbohydrates (Molisch test, Barford's test, Benedict's test), flavonoids (lead acetate test, alkaline reagent test), cardiac glycosides (Keller-Killiani test), anthraquinone glycosides (Borntrager's test), proteins and amino acids (Ninhydrin test), tannins and phenols (Ferric chloride test, lead acetate test, gelatin test), resins, saponins (Froth test), and terpenoids (Salkwoski's test) [22].

### 2.5. Antioxidant Potential of AME.Or

The potential of AME.Or to scavenge oxide free radicals was evaluated by the DPPH method [23] and total phenolic content (TPC) and total flavonoid content (TFC) by gallic acid equivalent and rutin equivalent method, respectively [24].

### 2.6. Experimental Animals

Male Wistar albino rats (*n* = 30) of either sex (180–220 g of body weight) were caged in the animal house of the Pharmacology research laboratory at the Department of Pharmacology, Faculty of Pharmacy, IUB. All of the animals used for the research were kept in polycarbonate cages of 4(7 × 34 × 18) cm^3^ dimensions, with a maximum of six animals per cage, under normal conditions of temperature (25 ± 2°C) and humidity (50–55%), as well as a 12 : 12 hour light and dark cycle. Animals were fed with a standard rodent diet and water *ad libitum*. The experiment's protocol was approved by the Institutional Animal Ethics Committee, Department of Pharmacology, Faculty of Pharmacy, IUB, and it was carried out in accordance with the committee's guidelines for the control and monitoring of animal studies under the AEC File No. PAEC/21/47.

### 2.7. *In Vivo* Study Design

Rats (*n* = 30) weighing 200 ± 20 g were divided into 5 groups (*n* = 6). All the rats were acclimatized for 2 weeks before the commencement of the experiments. The following treatments were provided to each of the six rats in each group over three weeks. Group A rats (normal control) received normal saline (5 ml/kg) through oral gavage for 3 weeks. Group B (intoxicated group) received CdCl_2_ prepared in normal saline (6.5 mg/kg) *via* an intraperitoneal route on the day 1^st^ of the study [25]. Group C rats (standard group) were administered with CdCl_2_ on day 1, followed by a treatment of silymarin (100 mg/kg via oral gavage) for 3 successive weeks [26]. Group D rats (treatment group 01) were administered orally with AME.Or at a dose of 200 mg/kg for 3 consecutive weeks and CdCl_2_ as in group B. Likewise, group *E* (treatment group 02) received an oral treatment of AME.Or (400 mg/kg) for 3 successive weeks and CdCl_2_ as in group B. Doses were carefully selected based on a previous study [27] at the same institute and previous lab experience with this plant.

On the 11^th^ day, the rats of all the groups were anesthetized using ketamine and xylaxine (10 : 1) at a 2 ml/kg dose, and their blood samples were collected by retro-orbital route for hematological analysis into labeled EDTA bottles to prevent clotting. Serum was collected into plain bottles and allowed to clot after which it was centrifuged at 4000 rpm for 15 minutes. The serum obtained was stored in a refrigerator at −4°C. On day 21, all the animals were fasted with a free supply of water for 24 h and sacrificed by the cervical dislocation method and serum was collected for hematological analysis. The liver was dissected from each rat and was kept in 10% formalin solution after weighing followed by the histopathological analysis.

### 2.8. Statistical Analysis

All the results were expressed as mean ± SEM of *n* = 6 in each group and statistically evaluated through Graphpad Prism 9.0.1 (La Jolla, CA, USA) to analyze the results by one-way analysis of variance (one-way ANOVA). Bonferroni's post hoc test was conducted to find the significance among various groups by setting *p* < 0.05.

## 3. Results

### 3.1. Phytochemical Screening of AME.Or

Phytochemical analysis revealed the presence of alkaloids, tannins, phenols, flavonoids, saponins, cardiac glycosides, and carbohydrates (Table 1). AMR.Or inhibited the DPPH free radicals scavenging up to 62.43 ± 2.72%. The TPC of the extract was found to be 79.92 ± 1.23 mg GAE/g. Likewise, the TFC of AMR.Or was 38.75 ± 0.94 mg RE/g.

### 3.2. The Effect of AME.Or on Liver Weight and Hepatic Functions

**Figure 1:**
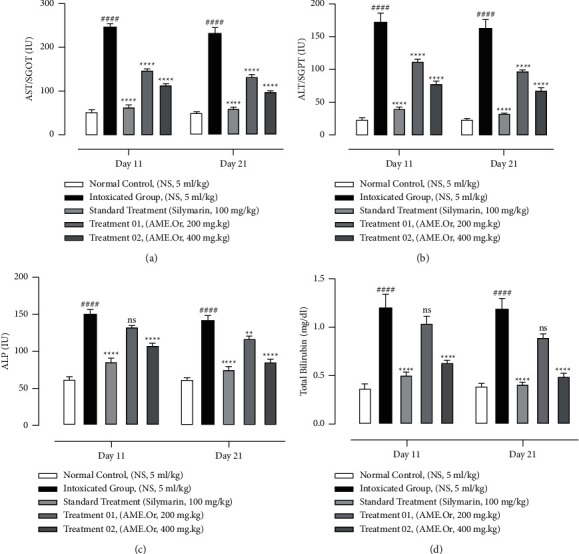
The effects of AME.Or on hepatic parameters. Cdcl_2_ was administered *via* the intraperitoneal route on the 1^st^ day of study followed by AME.Or doses at 200 mg/kg and 400 mg/kg. (a) AST/SGOT. (b) ALT/SGPT. (c) ALP. (d) Total Bilirubin was measured through the commercial kits method. Mean ± SEM of *n* = 6. Each group is analyzed using one-way ANOVA followed by Bonferroni's test. When the intoxicated group is compared to treatment groups, results are considered nonsignificant (ns) if *p* > 0.05, significant (^*∗*^) if *p* < 0.05, more significant (^*∗∗*^) if *p* < 0.01, very significant (^*∗∗∗*^) if *p* < 0.001, and highly significant (^*∗∗∗∗*^) if *p* < 0.0001. When the control group is compared with the intoxicated group, the significance of the results is denoted with (^#^). (ALT, alanine transaminase; AST, Aspartate transaminase; ALP, Alkaline phosphatase).

In the present study, the hepatic parameters, i.e., aspartate aminotransferase/serum glutamic-oxaloacetic transaminase (AST/SGOT), alanine aminotransferase/serum glutamic-pyruvic transaminase (ALT/SGPT), alkaline phosphatase (ALP) and total bilirubin were determined on the 11^th^ and 21^st^ day. The effect of different concentrations of AME.Or, i.e., 200 and 400 mg/kg, on the body and the liver weight is given in Table 2, whereas the hepatic parameters of the treated animals are shown in Figure 1. The Intoxicated group treated with CdCl_2_ showed a significant increase in hepatic parameters and AME.Or showed remarkable dose-dependent effects, i.e., normalization of hepatic function at a dose of 400 mg/kg p.o. And the results of treatment groups are almost comparable to the standard treatment (silymarin) group.

### 3.3. Lipid Profile (Cholesterol, Triglycerides, HDL, LDL, VLDL, AI)

In the present study, the lipid profile i.e. the total cholesterol, triglycerides, high-density lipoproteins (HDL), low-density lipoproteins (LDL), very-low-density lipoproteins (VLDL), and atherogenic index were estimated on the 11^th^ and 21^st^ day. The effect of different concentrations of AME.Or; i.e. 200 and 400 mg/kg, on different lipid parameters are shown in Table 3. The intoxicated group treated with CdCl_2_ showed marked imbalance (*p* ≤ 0.05) in lipids levels and AME.Or showed highly significant and dose-dependent effects, i.e., normalization of lipid profile at dose 400 mg/kg p.o., and the results of treatment groups were almost comparable with the silymarin-treated group.

### 3.4. The Effect of Various Treatments on the Liver Histopathology

**Figure 2:**
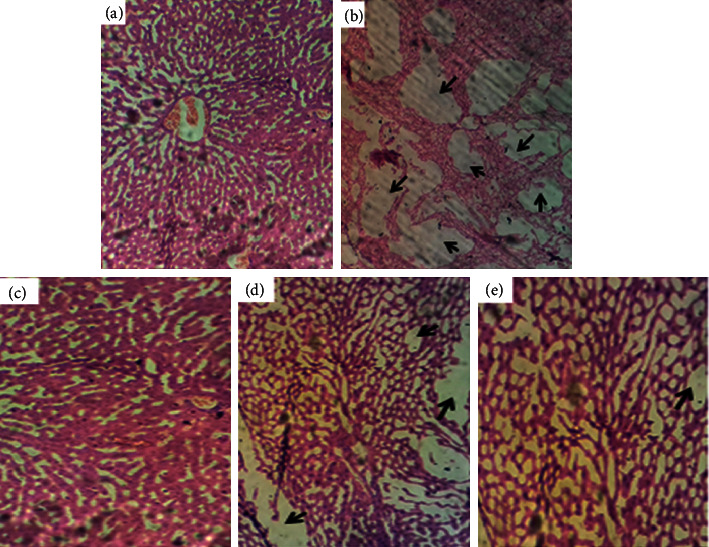
Histologic sections of the liver. (a) Normal control. (b) Intoxicated. (c) Silymarin, 100 mg/kg. (d) AME.Or, 200 mg/kg. (e) AME.Or, 400 mg/kg. ( ) Enlarged interstitial spaces of hepatic epithelium. Cdcl_2_ was administered *via* the intraperitoneal route on the 1^st^ day of study followed by AME.Or doses at 200 mg/kg and 400 mg/kg. Liver sections were processed for histopathological analysis after euthanization.

The integrity of hepatocytes and the histopathologic alterations after the 21-day treatment with AME. Or are presented in Figure 2. The intoxicated group showed prominent epithelial damage, enlarged endothelial spaces, disarrangement, and loss of characteristic integrity of the liver. Mild necrosis was observed across the lobules. AME.Or treatment prevented Cd-induced necrotic lesions in a dose-dependent manner. The hepatic tissue in the treatment groups appeared normal except few scattered necrotic areas.

## 4. Discussion

Cd is a toxic heavy metal listed on the priority list of hazardous wastes by USEPA (the United States Environmental Protection Agency) [28]. Because of industrial and agricultural activities, the Cd level in the environment is on a consistent rise [29]. The present study evaluated different hepatic serum enzymes (AST/SGOT, ALT/SGPT, ALP, and TB), considering their levels as a measure of hepatotoxicity [30]. Our findings affirmed that the hepatic cell integrity is disrupted as a result of Cd exposure with subsequent hepatic failure. Several animal investigations have also found elevated levels of serum hepatic enzymes in rats when administered CdCl_2_, confirming the current findings and emphasizing the impact of Cd exposure [26, 31–33]. Indeed, Cd-intoxication therapy has been investigating some natural phytoconstituents with hepatotonic effects [34]. In this research, *O. ramosa* (Roxb.) was studied to investigate its hepatotonic effects against Cd-induced hepatic injury with lipids imbalance.

In this study, the intoxicated group showed a significant decrease in body weight and an increase in liver weight. The alteration in body weight may be due to the dysfunction of the glucocorticoid system with impaired glucocorticoid hormones which are implicated in lipids metabolism [34, 35]. Another reason for weight loss may be an accumulation of Cd in the liver and other vital organs leading to functional damage and interstitial fibrosis [36]. In contrast to the intoxicated group, AME.Or treatment mitigated the Cd-induced weight loss and fatty liver. Although reported data on traditional medicinal activity data on *O. ramosa* (Roxb.) is scarce, few ethnobotanical studies highlight it in jaundice and gastrointestinal ailments [11, 37].

Hepatic biomarker enzyme levels, cholesterol, triglycerides, and lipoproteins levels are indicators of hepatotoxicity [21, 26, 35, 38]. The observed significant increase (*p* < 0.001) in the levels of biomarkers, i.e., AST/SGOT, ALT/SGPT, ALP, total cholesterol, LDL, VLDL, triglycerides, total bilirubin, and atherogenic index [39–41] and a significant decrease (*p* < 0.001) of HDL level in serum of Cd treated rats is the evidence of hepatotoxicity and hyperlipidemia [42]. The release of SGOT/AST and ALT/SGPT into the systemic circulation is triggered by hepatic damage [43] and remains key indicators of hepatotoxicity [44, 45]. Cd alters the cellular structure and subsequent peroxidation of membranes may cause lipids imbalance. Cd decreased HDL and raised LDL concentration that suggests the dysregulation of metabolic features along with deterioration enzyme biomarkers [21]. Current research findings revealed a significant reversal of serum biomarkers to normal in all treatment groups in a dose-dependent manner after administration of AME.Or. AME.Or administered at 400 mg/kg body weight showed significant improvement in liver functionality and moderates the damage caused by Cd-intoxication. Indeed the plant is traditionally used in liver diseases for centuries [46] and our findings correspond to this reputation.

The effect of AME.Or treatment on serum biomarkers can be traced to its antioxidant potential which was investigated by DPPH free radical scavenging potential, TPC, and TFC of the extract [47]. The antioxidative potential of plants containing flavonoids also affects lipid regulation and related immune regulations [48]. Correspondingly, our study showed antioxidative activity along with lipid control and protection against liver enzymes. The phytochemical analysis also revealed the presence of flavonoids among others that forms the basis for antioxidative activities [49] and lipid regulation for future studies. In addition to flavonoids, the phytochemical analysis also revealed the presence of alkaloids, flavonoids, and phenols that cause to mitigate the cytotoxic effects of intoxication-derived free radicals. These findings are in line with the previously published data on the plant [27].

*O. ramosa* (Roxb.) extract also possesses lipid-lowering and hepatotonic potential due to its phytochemical constituents i.e. flavonoids, terpenoids, tannins, saponins, and cardiac glycosides, which are previously reported for their physiologic actions on lipids metabolism [1, 21, 50].

## 5. Conclusion

The present study demonstrated the hepatoprotective potential of AME.Or against Cd-induced hepatotoxicity. Phytochemical screening of aqueous methanolic extract revealed the presence of alkaloids, tannins, phenols, flavonoids, saponins, and carbohydrates. Antioxidant potential of AME.Or was also investigated. AME.Or showed significant restoration of hepatic biomarkers and lipid profile indicators towards normal dose-dependently (200 and 400 mg/kg). Thus, the study suggested that *O. ramosa* (Roxb.) possesses hepatotonic potential which is due to its antioxidant effects. The study was conducted on the rat model of human exposure revealing that AME.Or influences metabolism and prevents abnormalities in hepatic and lipid markers. Therefore, it reflects the scientific ground for folkloric uses of the *O. ramosa* (Roxb.). Further investigation is needed to clarify the underlying protective mechanisms and active components of AME.Or.

## Figures and Tables

**Table 1:** The table names the phytoconstituents and the type of test used to perform the phytochemical screening of AME.Or. The final column marks the presence or absence of the constituents.

Phytoconstituents	Test	Remarks
Alkaloids	Dragendroff's test	+
	Mayer's test	+
	Hager's test	+
	Wagner's test	+

Fixed oils		−
Terpenoids	Salkwoski's test	+
Steroids	Salkwoski's test	−
Anthraquinone glycosides	Borntrager's test	−
Cardiac glycoside	Keller-Killiani test	+
Proteins and amino acids	Ninhydrin test	−

Tannins and phenols	Ferric chloride test	+
	Lead acetate test	+
	Gelatin test	+

Gums		−

Flavonoids	Lead acetate test	+
	Alkaline reagent test	+

Saponins	Froth test	+

Carbohydrates	Molisch test	+
	Barford's test	+
	Benedict's test	+

+ sign indicates presence and − sign shows the absence of phytoconstituents.

**Table 2:** The effect of Cdcl_2_ on the Body and the Liver Weight. Cdcl_2_ was administered *via* the intraperitoneal route on the 1^st^ day of the study followed by AME.Or doses at 200 mg/kg and 400 mg/kg. Each group data is presented as *g* ± SEM of *n* = 6 rats. When comparing the cadmium-intoxicated group to the control group, the results are deemed insignificant (ns) if *p* > 0.05, significant (^*∗*^) if *p* < 0.05, more significant (^*∗∗*^) if *p* < 0.01, very significant (^*∗∗∗*^) if *p* < 0.001, and highly significant (^*∗∗∗∗*^) if *p* < 0.0001. When the control group is compared with the intoxicated group, the significance of the results is denoted with (^#^).

Treatment Group	Weight of animals (g)			Liver weight (g)
	1^st^ day	11^th^ day	21^st^ day	
Control group	193.7 ± 3.65	205.2 ± 2.21	211.7 ± 2.74	5.230 ± 0.254
Intoxicated (cdcl_2_ 6.5 mg/kg)	235 ± 2.37	206.7 ± 2.56	147.3 ± 5.37	6.1954 ± 0.218^##^
Silymarin, 100 mg/kg	215 ± 3.71	201 ± 2.35	195.7 ± 2.26	5.407 ± 0.111^*∗∗∗*^
AME.Or, 200 mg/kg	221 ± 2.62	196.8 ± 3.40	168.8 ± 2.12	6.104 ± 0.379^ns^
AME.Or, 400 mg/kg	219 ± 3.3	202.3 ± 2.82	186 ± 2.12	5.905 ± 0.361^*∗*^

± indicates a margin of error.

**Table 3:** The Effect of AME.Or on Lipid Profile. Cdcl_2_ was administered *via* the intraperitoneal route on the 1^st^ day of study followed by AME.Or doses at 200 mg/kg and 400 mg/kg. The results are presented as mean ± SEM (*n* = 6); each group is analyzed using one-way ANOVA followed by Bonferroni's test. When the intoxicated group is compared to treatment groups, results are considered nonsignificant (ns) if *p* > 0.05, significant (^*∗*^) if *p* < 0.05, more significant (^*∗∗*^) if *p* < 0.01, very significant (^*∗∗∗*^) if *p* < 0.001, and highly significant (^*∗∗∗∗*^) if *p* < 0.0001. When the control group is compared with the intoxicated group, the significance of the results is denoted with (^#^).

Lipid profile	Groups	A (normal control)	B (cdcl_2_6.5 mg/kg)	C (silymarin 100 mg/kg)	D (AME.Or 200 mg/kg)	E (AME.Or 400 mg/kg)
	Days					
Total cholesterol (mg/dl)	11^th^ day	137.800 ± 3.584	241.67 ± 4.12^###^	174.167 ± 5.677^*∗∗∗*^	206.667 ± 5.149^*∗∗∗*^	181.167 ± 2.548^*∗∗∗*^
	21^st^ day	138.462 ± 5.284	233.50 ± 4.023^###^	144.167 ± 6.867^*∗∗∗*^	174.000 ± 4.258^*∗∗∗*^	150.500 ± 5.290^*∗∗∗*^
Triglycerides (mg/dl)	11^th^ day	65.667 ± 6.103	125.167 ± 4.012^###^	90.067 ± 3.562^*∗∗∗*^	106.167 ± 5.056^ns^	99.167 ± 3.961^*∗∗∗*^
	21^st^ day	65.500 ± 5.506	120.500 ± 4.478^###^	65.833 ± 6.041^*∗∗∗*^	90.550 ± 4.695^*∗∗∗*^	74.000 ± 6.836^*∗∗∗*^
HDL (mg/dl)	11^th^ day	54.833 ± 3.978	28.000 ± 3.141^###^	44.167 ± 6.887^*∗∗∗*^	36.500 ± 2.187^*∗*^	42.833 ± 5.212^*∗∗*^
	21^st^ day	54.500 ± 2.997	29.167 ± 2.971^###^	55.833 ± 3.870^*∗∗∗*^	46.167 ± 3.683^*∗*^	53.333 ± 2.459^*∗∗*^
LDL (mg/dl)	11^th^ day	69.833 ± 4.164	188.633 ± 6.972^###^	111.987 ± 9.575^*∗∗∗*^	148.933 ± 5.401^*∗∗*^	118.890 ± 3.913^*∗∗∗*^
	21^st^ day	70.862 ± 3.800	173.067 ± 3.932^###^	75.167 ± 7.698^*∗∗∗*^	105.000 ± 6.272^*∗∗*^	82.367 ± 3.194^*∗∗∗*^
VLDL (mg/dl)	11^th^ day	13.133 ± 1.221	25.033 ± 0.802^###^	18.013 ± 0.712^*∗∗∗*^	21.233 ± 1.011^*∗*^	21.233 ± 1.011^*∗∗*^
	21^st^ day	13.100 ± 1.101	24.100 ± 0.896^###^	13.167 ± 1.208^*∗∗∗*^	17.133 ± 1.010^*∗∗*^	14.800 ± 1.367^*∗∗∗*^
Atherogenic index	11^th^ day	1.325 ± 0.154	7.288 ± 1.012^###^	3.068 ± 0.734^*∗∗∗*^	4.195 ± 0.413^*∗*^	2.833 ± 0.213^*∗∗∗*^
	21^st^ day	1.332 ± 0.119	6.340 ± 0.810^###^	1.375 ± 0.183^*∗∗∗*^	2.375 ± 0.277^*∗∗∗*^	1.553 ± 0.064^*∗∗∗*^

± indicates a margin of error.

## Data Availability

The data can be obtained from the corresponding author upon request.
